# Comparing sexual dysfunction in cosmetic rhinoplasty candidates and normal population among married women in Shiraz, Iran: A case-control study

**DOI:** 10.18502/ijrm.v22i12.18064

**Published:** 2025-01-31

**Authors:** Ali Sahraian, Masoud Janipour, Atoosa Ebrahimi, Zohre Zareizadeh, Pardis Habibi, Amirhossein Babaei

**Affiliations:** ^1^Research Center for Psychiatry and Behavioral Sciences, Shiraz University of Medical Sciences, Shiraz, Iran.; ^2^Otolaryngology Research Center, Department of Otolaryngology, Shiraz University of Medical Sciences, Shiraz, Iran.; ^3^Department of Psychiatry, School of Medicine, Shiraz University of Medical Sciences, Shiraz, Iran.; ^4^Student Research Committee, Shiraz University of Medical Sciences, Shiraz, Iran.

**Keywords:** Sexual dysfunction, Psychological, Rhinoplasty, Cosmetic, Female.

## Abstract

**Background:**

Disturbed self-esteem and self-image can be found in body dysmorphic individuals, and sexual dysfunction is also more frequent among these individuals. Body image concerns may also contribute to the tendency to undergo cosmetic procedures, including rhinoplasty.

**Objective:**

This study aimed to compare sexual dysfunction in married women who were candidates for cosmetic rhinoplasty to a control group.

**Materials and Methods:**

This case-control study was conducted among 342 married women in Shiraz, Iran, from December 2021–2022. The case group included 192 cosmetic rhinoplasty candidates, and the control group comprised 150 women who were not candidates for cosmetic procedures; they were selected from the ear, nose, and throat outpatient department. Data on demographic features, psycho-social history, and quality of marital life were collected by face-to-face interviews. To evaluate their sexual performance, the participants filled out the female sexual function index questionnaire.

**Results:**

No significant difference was observed between rhinoplasty subjects and the control group in the female sexual function index (23.63 ± 3.6 vs. 23.19 ± 4.28, p = 0.43). The prevalence of sexual dysfunction among cosmetic rhinoplasty candidates and the control group was 75% and 76%, respectively (p = 0.60). No significant difference was observed in the sexual dysfunction domains between the groups.

**Conclusion:**

Our study indicated a significant sexual dysfunction among the studied population, but the difference was not statistically significant between the rhinoplasty and the control group.

## 1. Introduction

Sexual activity is an important aspect of life and plays an essential role in the feelings of success and happiness in married life (1, 2). Furthermore, sexual satisfaction is correlated with healthy aging, cardiovascular benefits, physical activity, lower pain sensitivity, and relaxation; it also significantly affects the quality of life (1, 3). Interpersonal communication skills, conflict resolution skills, sociocultural taboos, socioeconomic status, family responsibilities, number of children, frequency of sex, aging, infertility, genital tract surgeries, obesity, depression, anxiety, rheumatologic disorders, and other health conditions are among the factors that affect sexual satisfaction (4). The World Health Organization defines sexual health as the emotional, mental, physical, and social well-being related to sexuality (5). Sexual dysfunction in females is applied for pain disorders or any problems in sexual desire, arousal, lubrication, and orgasm (6, 7). According to the diagnostic and statistical manual of mental disorders, text revision, female sexual dysfunction is divided into sexual interest/arousal disorder, female orgasmic disorder, and genito-pelvic penetration pain disorder. Moreover, it classifies the disorders as other specified, unspecified, and substance/medication-induced sexual dysfunctions (8). Sexual dysfunction is associated with an increase in the occurrence of mental health disorders such as anxiety, as well as dissatisfaction with married life and divorce (9).

One of the factors that influence sexual performance is sexual self-esteem, which is one of the determinants of sexual self-image, and it has been proven that impaired sexual self-esteem is related to decreased sexual satisfaction (10). Disturbed self-esteem and self-image can also be found in body dysmorphic individuals, and sexual dysfunction is also more frequent among individuals with body dysmorphic disorder (11, 12).

In addition to the excessive role of social media in increasing cosmetic surgery tendency, psychiatric problems such as body dysmorphic disorder also contribute to the increased attempt for aesthetic rhinoplasty (13, 14). Body image concern is significant in candidates for cosmetic procedures, including rhinoplasty (15, 16). A previous study found an improvement in sexual self-esteem and sexual life following cosmetic surgeries (17). Although studies have shown an improvement in the sexual quality of life of women who undergo cosmetic rhinoplasty, their sexual function and sexual life satisfaction have not been compared to the normal population before their surgery.

Given the effect of women's sexual satisfaction on their quality of life and mental health, we aimed to compare the prevalence of women's sexual dysfunction in cosmetic rhinoplasty candidates with a control group.

## 2. Materials and Methods

### Study design 

This case-control study was conducted on 342 women from December 2021–2022 in Khalili and Dastgheib hospital affiliated with Shiraz University of Medical Sciences, Shiraz, Iran.

### Participant recruitment and sample size

In this study, we used the convenience sampling method. Cosmetic rhinoplasty candidates were selected as the case group. Our control group consisted of women who visited the ear, nose, and throat outpatient department and were not candidates for cosmetic procedures. The inclusion criteria were female gender, married women, age > 18 yr old, marriage duration ≥ 6 months, and no acute or chronic physical or mental disorders. The exclusion criteria were drug abuse, living far from their husband, and any other conditions that affect sexual relationships, including pregnancy and gynecologic problems during the past 6 months. The selected controls were matched with the case group for age.

### Data gathering

Demographic data, including age, duration of the marriage, education, history of cigarette smoking, number of children, marital satisfaction, emotional relationship with husband, and the number of their sexual intercourse per month were gathered by face-to-face interview. The Persian-translated and validated female sexual function index (FSFI) questionnaire consisting of 19 questions was used to evaluate the female sexual function in different dimensions, including sexual desire, arousal, lubrication, orgasm, satisfaction, and sexual pain (18). The scores range from 2–36, and a higher score indicates better sexual function. An overall score below 28 is considered sexual dysfunction. The cut-off points for each dimension (sexual desire, arousal, lubrication, orgasm, satisfaction, and sexual pain) are 3.3, 3.4, 3.7, 3.4, 3.8, and 3.8, respectively (19).

### Ethical Considerations

The institutional review board of Shiraz University of Medical Sciences, Shiraz, Iran approved the study protocol. We also obtained the approval of the university Ethics Committee (Code: IR.SUMS.MED.REC.1400.426). Written informed consent was obtained from all participants.

### Statistical Analysis

In this study, the data of the numerical variables are shown as the mean ± standard deviation (SD), and the categorical variables are shown in percentages and frequencies. We used independent *t* test, Mann-Whitney, and Chi-square tests to evaluate the correlation between the variables. The Statistical Package for the Social Sciences (SPSS) software, version 23 (SPSS Inc. Chicago, IL), was used for performing the statistical analyses. A p-value < 0.05 was considered statistically significant.

## 3. Results

From a total of 353 participants, 11 out of the 203 female cosmetic rhinoplasty candidates were excluded from this study, and the final number of participants was 342. The rhinoplasty and control groups comprised 192 and 150 married women, respectively. No significant differences were found between the groups in age, marriage duration, education, financial status, number of children, and number of sexual intercourse per month (Table I). Marital satisfaction and emotional connection with their spouse were not significantly different between the groups (Table II).

The mean ± SD of FSFI in the study population, case, and control groups were 23.43 ± 3.92, 23.63 ± 3.6, and 23.19 ± 4.28, respectively. No statistically significant differences were found between the mean scores of the rhinoplasty and the control group in terms of sexual dysfunction, sexual desire, arousal, lubrication, orgasm, satisfaction, and sexual pain (Table III). Lubrication was the dimension that caused the highest dysfunction rates among the individuals (Table IV).

The mean duration of marriage in the sexual dysfunction group was significantly higher than the group with normal sexual function (p= 0.01). The number of intercourses per month was significantly higher in the group with normal sexual function than in the sexual dysfunction group (p < 0.01). The differences between these 2 groups regarding educational status, financial status, number of children, and marital satisfaction were also statistically significant (Table V).

**Table 1:** Demographic characteristics of the participants

**Characteristic**		**Case group (n = 192)**	**Control group (n = 150)**	**P-value**
Age (yr)*		33.91 ± 6.75 (34.5, 10)	34.38 ± 7.05 (33, 10)	0.77
Marriage duration (yr)*		11.9 ± 6.85 (10, 9.75)	12.18 ± 8.27 (10, 11.38)	0.69
Intercourses per month*		6.42 ± 4.29 (6, 4)	6.32 ± 4.85 (5, 5)	0.38
History of cigarette smoking**		2 (1.05)	8 (5.33)	0.02
Educational status**				
	**Below diploma**	21 (10.99)	19 (12.67)	0.06
	**Diploma**	64 (33.51)	41 (27.33)	
	**Associate degree**	14 (7.33)	26 (17.33)	
	**Bachelor's and Master's degree**	87 (45.55)	61 (40.67)	
	**Doctorate and above**	6 (3.14)	3 (2.00)	
Financial status**				
	**Poor**	4 (2.09)	10 (6.67)	0.16
	**Intermediate**	105 (54.97)	76 (50.67)	
	**Good**	78 (40.84)	58 (38.67)	
	**Excellent**	5 (2.62)	6 (4.00)	
Number of children**				
	**0**	31 (16.23)	34 (22.67)	0.29
	**1–2**	149 (78.01)	106 (70.67)	
	**3–5**	12 (6.28)	9 (6.00)	
	**> 5**	0 (0.00)	1 (0.67)	
*Data presented as Mean ± SD (median, interquartile range), Independent *t* test. **Data presented as n (%), Chi-square test				

**Table 2:** The level of marital satisfaction and emotional connection with the husband

**Characteristic**		**Case group (n = 192)**	**Control group (n = 150)**	**P-value**
**Marital satisfaction**				
	**Very satisfied**	95 (49.48)	77 (51.33)	0.45
	**Moderately satisfied**	78 (40.63)	55 (36.67)	
	**Slightly satisfied**	14 (7.29)	8 (5.33)	
	**Not satisfied**	5 (2.60)	10 (6.67)	
**Satisfaction from husband**				
	**Very satisfied**	106 (55.21)	76 (50.67)	0.25
	**Moderately satisfied**	70 (36.46)	58 (38.67)	
	**Slightly satisfied**	14 (7.29)	11 (7.33)	
	**Not satisfied**	2 (1.04)	5 (3.33)	
**Satisfaction from an emotional relationship with the spouse**				
	**Very satisfied**	107 (55.73)	73 (48.67)	0.11
	**Moderately satisfied**	64 (33.33)	59 (39.33)	
	**Slightly satisfied**	18 (9.38)	10 (6.67)	
	**Not satisfied**	3 (1.56)	8 (5.33)	
Data presented as n (%), Chi-square test				

**Table 3:** Comparison of sexual function and its dimensions

**Characteristic**	**Case group (n = 192)**	**Control group (n = 150)**	**P-value**
**FSFI**	23.63 ± 3.6	23.19 ± 4.28	0.43
**Sexual desire**	3.82 ± 0.96	3.74 ± 1.05	0.43
**Arousal**	4.07 ± 1.09	3.93 ± 1.22	0.30
**Lubrication**	3.17 ± 1.23	3.15 ± 0.85	0.66
**Orgasm**	3.63 ± 0.7	3.55 ± 0.81	0.18
**Satisfaction**	4.71 ± 1.11	4.49 ± 1.15	0.06
**Sexual pain**	4.08 ± 1.36	4.1 ± 1.39	0.53
Data presented as Mean ± SD, Independent samples *t* test. FSFI: Female sexual function index			

**Table 4:** Comparison of the prevalence of people with sexual dysfunction and its dimensions between the participants with normal sexual function and sexual dysfunction

**Characteristic**	**Case group (n = 192)**	**Control group (n = 150)**	**P-value**
**FSFI**	144 (75.0)	114 (76.0)	0.60
**Sexual desire**	41 (21.4)	40 (26.7)	0.25
**Arousal**	41 (21.4)	38 (25.3)	0.38
**Lubrication**	173 (90.1)	129 (86.0)	0.24
**Orgasm**	51 (26.5)	50 (33.3)	0.16
**Satisfaction**	38 (19.8)	33 (22.0)	0.61
**Sexual pain**	75 (39.1)	61 (40.7)	0.92
Data presented as n (%), Chi-square test. FSFI: Female sexual function index			

**Table 5:** Comparison of the variables between the participants with normal sexual function and sexual dysfunction

**Characteristic**		**Sexual dysfunction (n = 271)**	**Normal sexual function (n = 71)**	**P-value**
Age (yr)*		34.30 ± 6.78	33.19 ± 6.54	0.25
Marriage duration (yr)*		12.55 ± 7.59	9.42 ± 6.45	< 0.01
Intercourses per month*		6.05 ± 4.44	7.93 ± 5.03	< 0.01
History of cigarette smoking**		7 (2.58)	2 (2.81)	0.91
Educational status**				
	**Below diploma**	36 (13.28)	3 (4.23)	0.02
	**Diploma**	83 (30.63)	20 (28.17)	
	**Associate degree**	33 (12.18)	8 (11.27)	
	**Bachelor's and master's degree**	115 (42.44)	35 (49.30)	
	**Doctorate and above**	4 (1.48)	5 (7.04)	
Financial status**				
	**Poor**	14 (5.17)	0 (0.00)	0.01
	**Intermediate**	152 (56.09)	29 (40.85)	
	**Good**	98 (36.16)	36 (50.70)	
	**Excellent**	7 (2.58)	6 (8.45)	
Number of children**				
	**0**	46 (16.97)	17 (23.94)	< 0.01
	**1–2**	207 (76.38)	36 (50.70)	
	**3–5**	17 (6.27)	17 (23.94)	
	**> 5**	1 (0.37)	1 (1.41)	
Marital satisfaction**				
	**Very satisfied**	127 (46.86)	46 (64.79)	0.02
	**Moderately satisfied**	110 (40.59)	23 (32.39)	
	**Slightly satisfied**	22 (8.12)	2 (2.82)	
	**Not satisfied**	12 (4.43)	0 (0.00)	
Satisfaction from husband**				
	**Very satisfied**	134 (49.45)	51 (71.83)	0.01
	**Moderately satisfied**	108 (39.85)	19 (26.76)	
	**Slightly satisfied**	23 (8.49)	1 (1.41)	
	**Not satisfied**	6 (2.21)	0 (0.00)	
Satisfaction from an emotional relationship with the spouse**				
	**Very satisfied**	129 (47.60)	53 (74.65)	< 0.01
	**Moderately satisfied**	106 (39.11)	16 (22.54)	
	**Slightly satisfied**	27 (9.96)	1 (1.41)	
	**Not satisfied**	9 (3.32)	1 (1.41)	
*Data presented as Mean ± SD, Chi-square test. **Data presented as n (%), Chi-square test				

## 4. Discussion

In our study, sexual dysfunction was found in 75.4% of the individuals. Lubrication was the most problematic dimension of sexual dysfunction and affected 88.3% of the participants. No significant difference was observed between the rhinoplasty and the control group regarding the prevalence of sexual dysfunction and its domains. In line with our findings, another study found that the prevalence of sexual dysfunction in Iranian women was 77.6% (20). The mean score of FSFI in women seeking radiofrequency of external genitalia was 25.41 ± 6.34, showing a lower sexual dysfunction than our study participants (21). In a systematic review and meta-analysis that included 7 original articles, 52% of the studied women suffered from sexual dysfunction. The prevalence of each sexual dysfunction domain, including sexual desire, arousal, lubrication, pain, and orgasm was 39%, 34%, 32%, 38%, and 30%, respectively. In contrast to our study results, decreased sexual desire and pain were more prevalent than the other dimensions of sexual dysfunction (22). In another study, the prevalence of sexual dysfunction among women was 31%, and the prevalence rates of decreased sexual desire, arousal, orgasm, and dyspareunia were 33%, 16.5%, 25%, and 45.5%, respectively. Hence, their findings were different from ours in terms of sexual dysfunction prevalence and the most prevalent domain of sexual dysfunction. They also found a statistically significant relationship between women's sexual dysfunction and marriage duration, history of mental disorder, and psychiatric drug consumption. These results are in line with those of our study in that we found a statistically significant relationship between the duration of marriage and sexual dysfunction. They also declared that educational status was not correlated to sexual dysfunction (23).

A study on women suffering from diabetes and hypertension in South Africa found that 94% of the sexually active women had an FSFI score < 26.55 and were considered to have sexual dysfunction. Lack of desire and sexual pain were observed in 90% and 83% of them, respectively, and all of them had lubrication and orgasm problems. Their underlying chronic diseases can be justified by the high prevalence of sexual dysfunction (24). In another study which was conducted on Singaporean women, 70.3% of the sexually active cases met the criteria for female sexual dysfunction. As opposed to the findings of our study, sexual dysfunction was positively associated with nulliparity (25). In a study that was conducted on Indian women, 63% of women reported desire dysfunction, 77% suffered arousal disorder, 51% had lubrication problems, and 56% had sexual pain. These higher dysfunction rates can be attributed to their different questionnaire structure (26).

In our study, marital satisfaction and emotional connection with the husband were related to decreased sexual dysfunction. It is known that sexual desire in women is highly sensitive to emotional intimacy with their partners. This condition may also make women the initiators of the sexual relationship, and they experience more pleasure in such situations (27). It seems that inadequate knowledge and feeling ashamed to talk about sexual issues are among the factors that contribute to the occurrence of sexual problems in Iranian women (28).

Various conditions such as diabetes, depression, intimate partner violence, and specific drug consumption contribute to female sexual dysfunction. Due to the numerous aetiologies of sexual dysfunction, a complete history should be taken, and a physical examination must be done. Based on the etiology inferred from the history taking and physical examination, education, medication therapy, psychological interventions, and physical therapy would be some of our therapeutic options (29). Regarding the significant prevalence of female sexual dysfunction, it is suggested that screening should be added to routine checkups (30).

Our study had some limitations. We can point out the lack of examination of body image concerns and sexual self-esteem. The availability of such data would let us investigate the relationship of sexual dysfunction with sexual self-esteem and body dysmorphic disorder among the study groups.

## 5. Conclusion

In conclusion, this study found that there was no significant difference in sexual dysfunction between married women who were candidates for cosmetic rhinoplasty and those in the control group. The prevalence of sexual dysfunction was high in both groups, highlighting the importance of searching for its underlying causes. In our research, we found a statistically significant relationship between sexual dysfunction and the number of children; socio-economic status and duration of marriage also affected sexual function.

Further research is needed to explore the impact of sexual orientation, body image concerns, and other mental health issues on the sexual function of women.

##  Data Availability

Data supporting the findings of this study are available upon reasonable request from the corresponding author.

##  Author Contributions

A. Sahraian: Concept and design, critical revision of the manuscript, final approval of the version to be published, agreement to be accountable for all aspects of the work. M. Janipour: Concept and design, critical revision of the manuscript, final approval of the version to be published, and agreement to be accountable for all aspects of the work. A. Ebrahimi: Acquisition of data, drafting of the manuscript, final approval of the version to be published; agreement to be accountable for all aspects of the work. Z. Zareizadeh: Acquisition of data, drafting of the manuscript, final approval of the version to be published; agreement to be accountable for all aspects of the work. P. Habibi: Statistical analysis, drafting of the manuscript, final approval of the version to be published; agreement to be accountable for all aspects of the work. A. Babaei: Statistical analysis, drafting of the manuscript, final approval of the version to be published; agreement to be accountable for all aspects.

##  Conflict of Interest

The authors have no conflict of interest to disclose.
